# Comparison of three amplicon sequencing approaches to determine staphylococcal populations on human skin

**DOI:** 10.1186/s12866-021-02284-1

**Published:** 2021-07-28

**Authors:** Charlotte Marie Ahle, Kristian Stødkilde-Jørgensen, Anja Poehlein, Wolfgang R. Streit, Jennifer Hüpeden, Holger Brüggemann

**Affiliations:** 1 Beiersdorf AG, Research & Development, Front End Innovation, 20245 Hamburg, Germany; 2 Department of Microbiology and Biotechnology, University of Hamburg, 22609 Hamburg, Germany; 3 Department of Biomedicine, Aarhus University, 8000 Aarhus, Denmark; 4 Department of Genomic and Applied Microbiology, Institute of Microbiology and Genetics, University of Göttingen, 37073 Göttingen, Germany

## Abstract

**Background:**

Staphylococci are important members of the human skin microbiome. Many staphylococcal species and strains are commensals of the healthy skin microbiota, while few play essential roles in skin diseases such as atopic dermatitis*.* To study the involvement of staphylococci in health and disease, it is essential to determine staphylococcal populations in skin samples beyond the genus and species level. Culture-independent approaches such as amplicon next-generation sequencing (NGS) are time- and cost-effective options. However, their suitability depends on the power of resolution.

**Results:**

Here we compare three amplicon NGS schemes that rely on different targets within the genes *tuf* and *rpsK,* designated tuf1, tuf2 and rpsK schemes. The schemes were tested on mock communities and on human skin samples. To obtain skin samples and build mock communities, skin swab samples of healthy volunteers were taken. In total, 254 staphylococcal strains were isolated and identified to the species level by MALDI-TOF mass spectrometry. A subset of ten strains belonging to different staphylococcal species were genome-sequenced. Two mock communities with nine and eighteen strains, respectively, as well as eight randomly selected skin samples were analysed with the three amplicon NGS methods. Our results imply that all three methods are suitable for species-level determination of staphylococcal populations. However, the novel tuf2-NGS scheme was superior in resolution power. It unambiguously allowed identification of *Staphylococcus saccharolyticus* and distinguish phylogenetically distinct clusters of *Staphylococcus epidermidis*.

**Conclusions:**

Powerful amplicon NGS approaches for the detection and relative quantification of staphylococci in human samples exist that can resolve populations to the species and, to some extent, to the subspecies level. Our study highlights strengths, weaknesses and pitfalls of three currently available amplicon NGS approaches to determine staphylococcal populations. Applied to the analysis of healthy and diseased skin, these approaches can be useful to attribute host-beneficial and -detrimental roles to skin-resident staphylococcal species and subspecies.

**Supplementary Information:**

The online version contains supplementary material available at 10.1186/s12866-021-02284-1.

## Background

Studying the skin microbiome is regarded increasingly important in understanding skin diseases as well as skin health. The genus *Staphylococcus* is one of the most abundant bacterial genera in the human skin microbiome; it plays a central role on human skin and in health and disease [1–17]. While the skin colonization by *Staphylococcus aureus* is correlated with disease severity, as seen for example in atopic dermatitis [1], coagulase-negative staphylococci (CoNS) are regarded as having rather health-beneficial roles on human skin. Common CoNS species that can be found on human skin are *Staphylococcus epidermidis, Staphylococcus hominis, Staphylococcus capitis* and *Staphylococcus haemolyticus* and others [18, 19]. As an important host-beneficial mechanism of certain CoNS, colonization resistance can prevent the expansion of potential pathogens on the skin; this is achieved by different CoNS properties such as the production of bacteriocins and phenol-soluble modulins [2–5] and quorum-sensing interference [6, 7]. Other host beneficial mechanisms of CoNS include, for example, the training and fortification of skin immunity [8–11], supporting wound healing [12, 13], and, possibly, anti-cancer effects [14]. Such host-interacting functions of CoNS are often species-, subspecies-, phylotype- and even strain-specific [2, 11, 14, 20, 21].

It was shown that one individual is not only colonised by an array of different staphylococcal species, but also by different strains of each species, in particular of *S. epidermidis* [22, 23]. The population of *S. epidermidis* species consists of strains belonging to three main phylogenetically distinct clades (A, B and C) [24–26]. In addition, a myriad of individual strains within each clade can be distinguished that differ in the core genome by single nucleotide polymorphisms (SNPs) and in the flexible genome by strain-specific genomic islands and extrachromosomal plasmids [20, 24, 25, 27].

To comprehensively map staphylococci on human skin, specific methods are needed to determine populations beyond the genus and species level. Traditionally, studies concerning the determination of staphylococci employed cultivation approaches with solid agar-based media [28, 29]. This makes it possible to investigate the isolated strains regarding their geno- and phenotypes. Depending on the choice of media and growth conditions, cultivation methods can underrepresent slow-growing and fastidious microorganisms. In contrast, culture-independent methods employing next-generation sequencing (NGS) achieve a more comprehensive picture of the skin microbiome [30]. Previous culture-independent studies have often relied on the 16S rRNA gene. However, this gene is inadequate to sufficiently distinguish several different staphylococcal species, and does not discriminate populations beyond the species level [31, 32]. Alternative target genes for identification and differentiation of staphylococcal isolates were evaluated and proposed such as *kat* [33], *gap* [31, 34, 35]*, hsp60* [36, 37]*, rpoB* [38, 39] and *sodA* [40]*.* One of the most established gene targets is *tuf*, which codes for elongation factor Tu (EF-Tu) [41]. The *tuf* gene, or rather fragments thereof, is also used as a target for analysing mixed staphylococcal communities with NGS methods, hereafter referred to as amplicon NGS [42–45]. Furthermore, the staphylococcal *rpsK* gene that encodes the 30S ribosomal protein S11 was recently proposed for amplicon NGS [46].

Here, we first isolated staphylococcal strains obtained from skin swabs of healthy volunteers, in order to assemble staphylococcal mock communities. We then compared three amplicon NGS schemes for their suitability to determine the staphylococcal populations of these mock communities. Two tested amplicon NGS schemes target different *tuf* gene fragments; one was developed by Martineau et al. [41] and the other one by Ahle et al. [47], designated here tuf1 and tuf2 scheme, respectively. The third tested scheme, designated rpsK scheme, targets a fragment of the *rpsK* gene and was developed by Ederveen et al. [46]. Lastly, the three amplicon NGS schemes were tested on skin swab samples obtained from healthy volunteers.

## Results

### Origin of targets for amplicon NGS

**Fig. 1:**
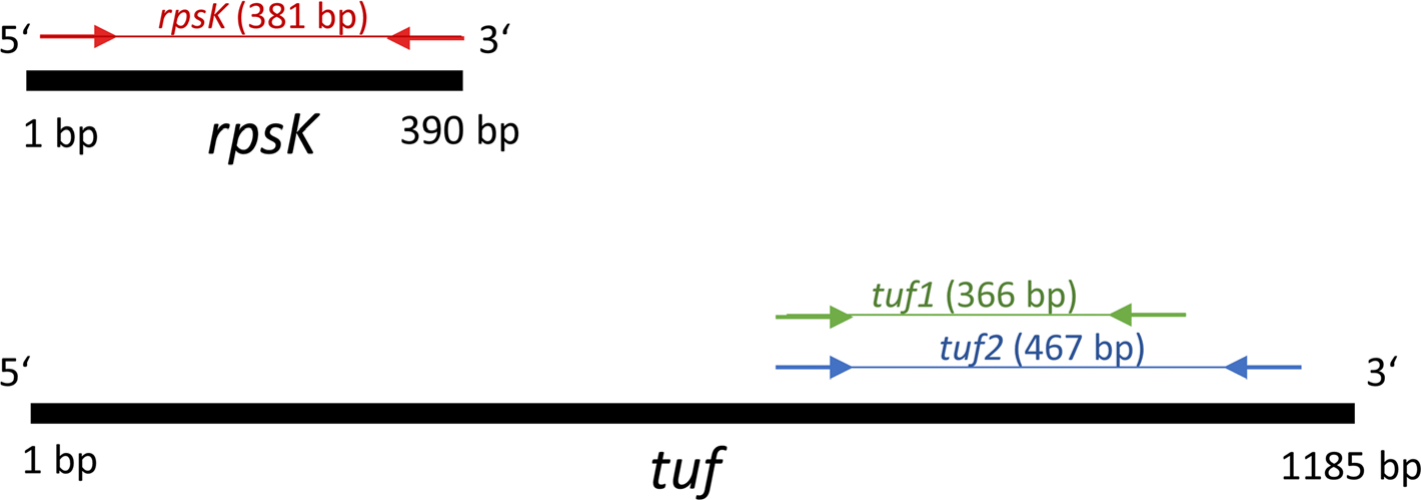
Location of amplicon targets for the three schemes to determine staphylococcal populations. The amplified region of the *rpsK* and *tuf* genes for the three schemes is shown (rpsk = red, tuf1 = green, tuf2 = blue)

Here, we compared three different amplicon NGS schemes, all previously published and designed for determining staphylococcal populations in mixed communities: tuf1 and tuf2 schemes target the *tuf* gene and the rpsK scheme targets the *rpsK* gene. The rpsK and tuf1 amplicon targets have a similar length with 381 and 366 bp, respectively, while the tuf2 amplicon target is with 467 bp the longest among the three targets (Fig. 1). The amplicon targets tuf1 and tuf2 overlap to some extent (position 688 to 1053 bp and 685 to 1151 bp, respectively).

### Amplicon NGS of bacterial mock communities

Two different bacterial mock communities (M1/M2) were prepared, containing DNA of nine (M1) and 18 (M2) different staphylococcal strains, respectively (Additional file 1). All utilized strains belonged to staphylococcal species commonly found on human skin. They originated either from publicly accessible collections or were isolated here from healthy skin. Strains isolated here, in total 254 strains, were first identified to the species level by MALDI-TOF mass spectrometry (Additional file 2). A subset of ten strains belonging to different staphylococcal species were subsequently genome sequenced to obtain a complete genome database for the two mock communities (Additional file 3).

**Fig. 2:**
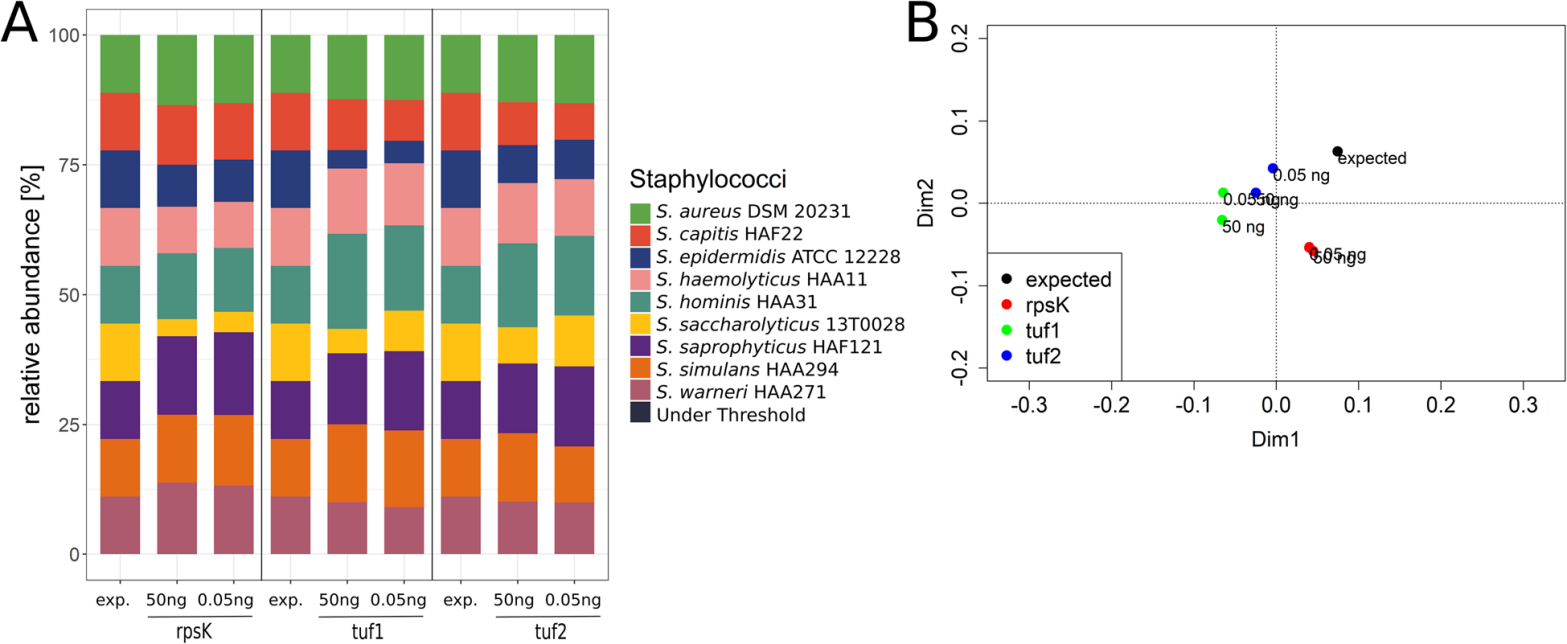
Relative abundancies of staphylococcal species within the mock community M1, determined by three different amplicon NGS schemes. Relative abundancies of staphylococcal species in the mock community M1 were determined with the rpsK, tuf1, and tuf2 schemes. Two DNA input amounts (50 ng and 0.05 ng per strain) were used. A) Stacked bar plots of relative abundances of mock community M1 samples. B) PCoA of Bray Curtis dissimilarity of the expected sample composition (=black) and mock community M1 analysed with the rpsK (=red), tuf1 (=green) and tuf2 (=blue) schemes

To test whether the three NGS schemes can distinguish between staphylococci on species level, the mock community M1 was analysed. The mock community M1 contained one strain each of *S. aureus, S. capitis, S. epidermidis*, *S. haemolyticus, S. hominis, Staphylococcus saccharolyticus, Staphylococcus saprophyticus, Staphylococcus simulans* and *Staphylococcus warneri* (Additional file 1). DNA was pooled in equimolar ratios and the three amplicon NGS pipelines were applied*.* All three schemes were able to identify and distinguish each of the nine species. The rpsK and the tuf1 schemes slightly underrepresented *S. saccharolyticus* and *S. epidermidis,* respectively (Fig. 2A)*.* A principal coordinate analysis (PCoA) plot of Bray Curtis dissimilarity was constructed to examine how accurate each scheme can represent the expected staphylococcal composition of mock community M1. The tuf2 scheme represented the expected sample composition more accurately than the other two schemes. We repeated the experiment with different DNA input amounts, varying from 0.05 ng to 50 ng DNA per strain. The DNA input amount did only mildly influence the detected relative abundancies by the three schemes (Fig. 2 A and B).

The second mock community M2 was composed of 18 strains to investigate whether the schemes were able to resolve diversity of samples beyond the species level. The mock community M2 included genomic DNA of one *S. aureus* strain*,* two *S. capitis* strains*,* four *S. epidermidis* strains, two *S. haemolyticus* strains*,* three *S. hominis* strains*,* two *S. saccharolyticus* strains*,* one *S. saprophyticus* strain*,* one *S. simulans* strain and two *S. warneri* strains (Additional file 1).

First, we calculated the theoretical resolution power of each scheme regarding the M2 community. To do so, we extracted the three target alleles of each of the 18 genomes present in M2 and built phylogenetic trees. The trees showed that the rpsK, tuf1 and tuf2 schemes should distinguish 12, 14 and 15 strains, respectively (Additional file 4). Thus, in silico, the tuf2 scheme is superior in resolution power.

**Fig. 3:**
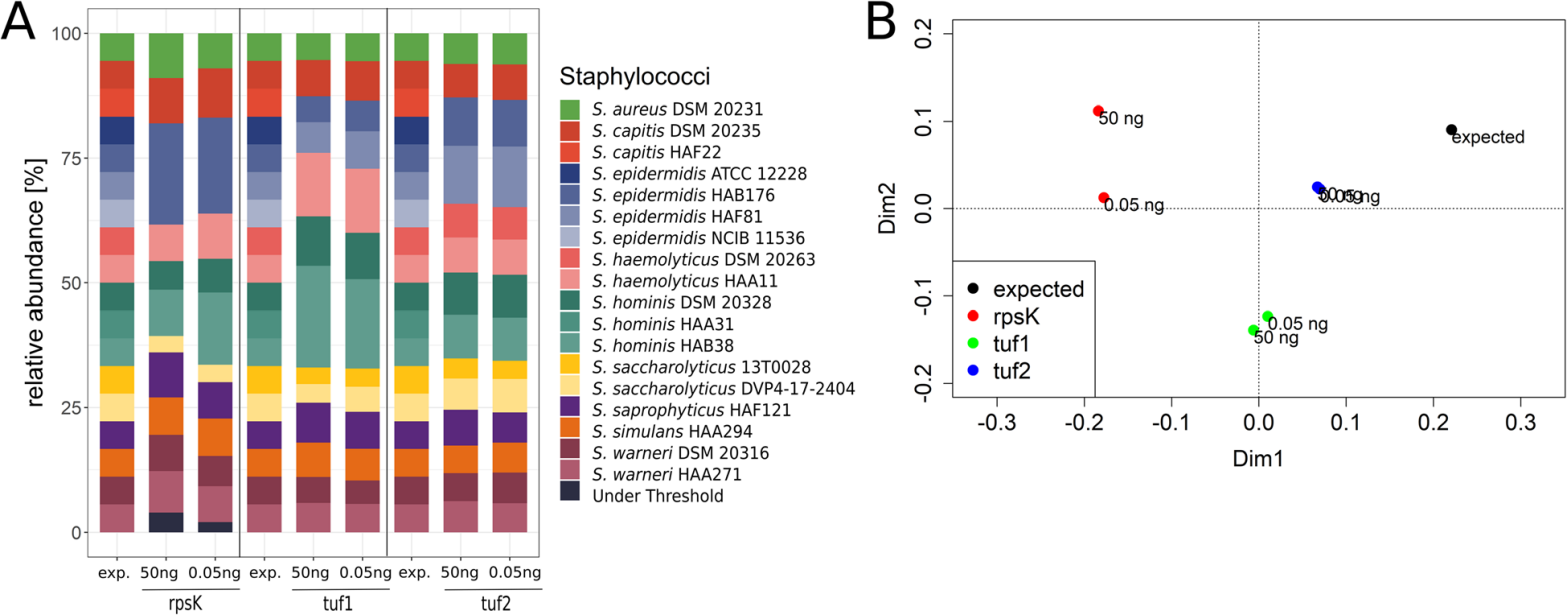
Relative abundancies of staphylococcal species within the mock community M2, determined by three different amplicon NGS schemes. Relative abundancies of staphylococcal species in the mock community M2 were determined with the rpsK, tuf1, and tuf2 schemes. Two DNA input amounts (50 ng and 0.05 ng per strain) were used. A) Stacked bar plots of relative abundances of mock community M2 samples. B) PCoA of Bray Curtis dissimilarity of the expected sample composition (=black) and mock community M2 analysed with the rpsK (=red), tuf1 (=green) and tuf2 (=blue) schemes

Next, we applied the three schemes to analyse the DNA cocktail of the mock community M2. The rpsK scheme detected 11 strains, while the tuf1 and tuf2 schemes detected 13 and 14 strains, respectively (Fig. 3A). The PCoA plot from Bray Curtis dissimilarity showed that the expected sample composition was best reflected by the data generated with the tuf2 scheme. Furthermore, compared to the two tuf schemes, data generated with the rpsK scheme showed a higher divergence when different DNA input amounts were used (Fig. 3 A and 3B).

### Amplicon NGS of human skin swab samples

Next, we applied the three schemes for the determination of staphylococcal populations in vivo. Eight skin swab samples from eight different volunteers were randomly selected. These eight samples included two samples from each of the four skin sites investigated (forehead, cheek, forearm, back).

**Fig. 4:**
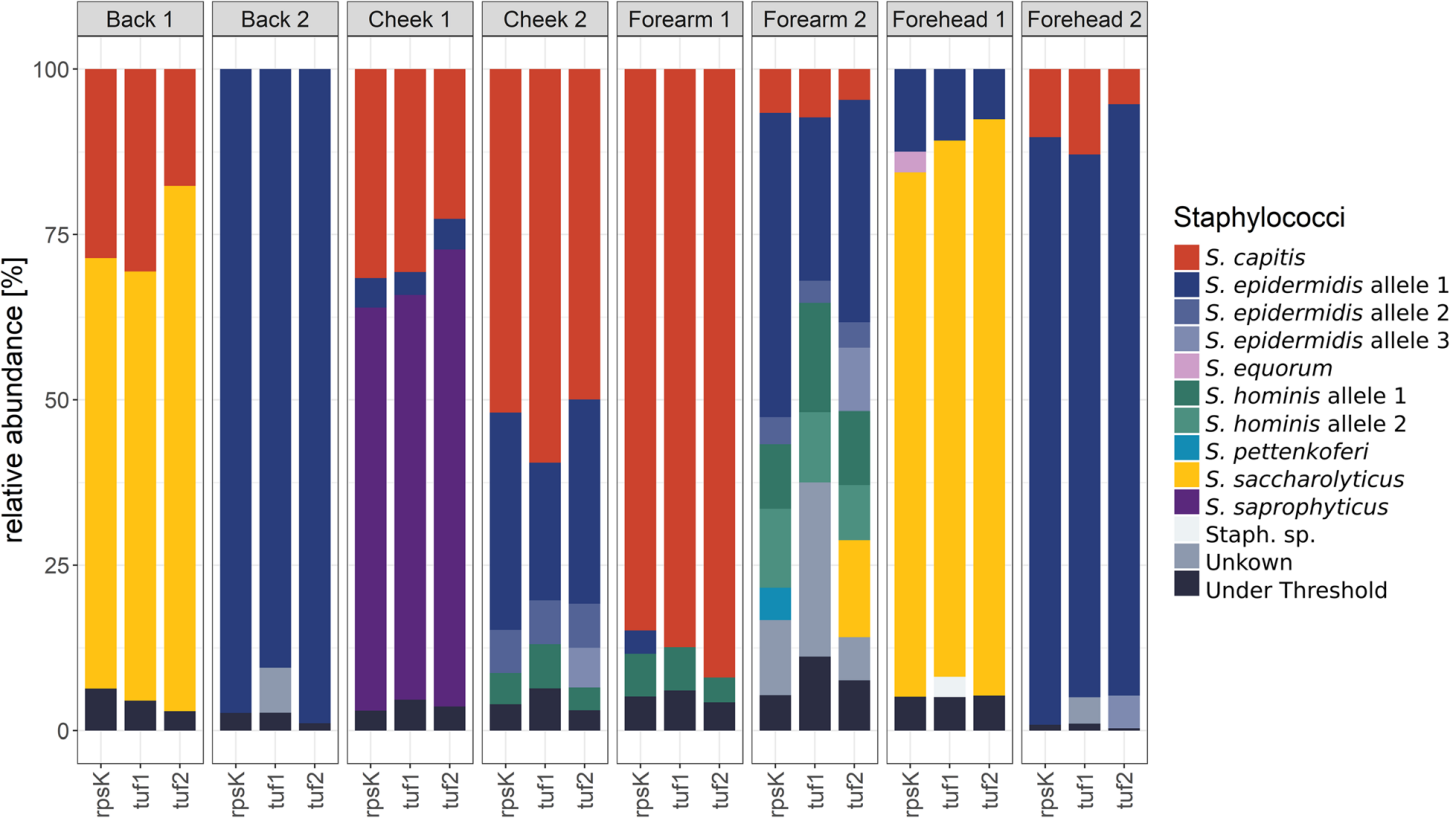
Staphylococcal composition of in vivo skin swab samples analysed with three different amplicon NGS schemes. The relative abundance of staphylococci in eight different samples (two samples of the four skin areas back, cheek, forearm and forehead) were analysed with three amplicon NGS schemes (rpsK/tuf1/tuf2). The three schemes determined highly similar populations, with differences in the detection of *S. saccharolyticus* and on the sub-species level

Overall, all three schemes detected a similar staphylococcal species composition in the analysed skin swab samples (Fig. 4). The most prominent differences were seen in one of the forearm samples (“forearm 2”); here, the tuf2 scheme detected *S. saccharolyticus*, whereas the rpsK and tuf1 schemes did not. Moreover, small differences were noted: first, the rpsK scheme detected *S. epidermidis* and *Staphylococcus pettenkoferi* in forearm samples, and *Staphylococcus equorum* in a forehead sample (“forehead 1”), all of which with low relative abundancies; in contrast, these three species were not detected by the two tuf schemes. Second, the tuf2 scheme detected three different *S. epidermidis* alleles in one cheek (“cheek 2”) and one forearm sample (“forearm 2”). In addition, in one forehead sample (“forehead 2”), the tuf2 scheme found two different *S. epidermidis* alleles. In contrast, the other two schemes detected one *S. epidermidis* allele less in each sample.

### In silico comparison of *S. epidermidis* amplicon targets

**Fig. 5:**
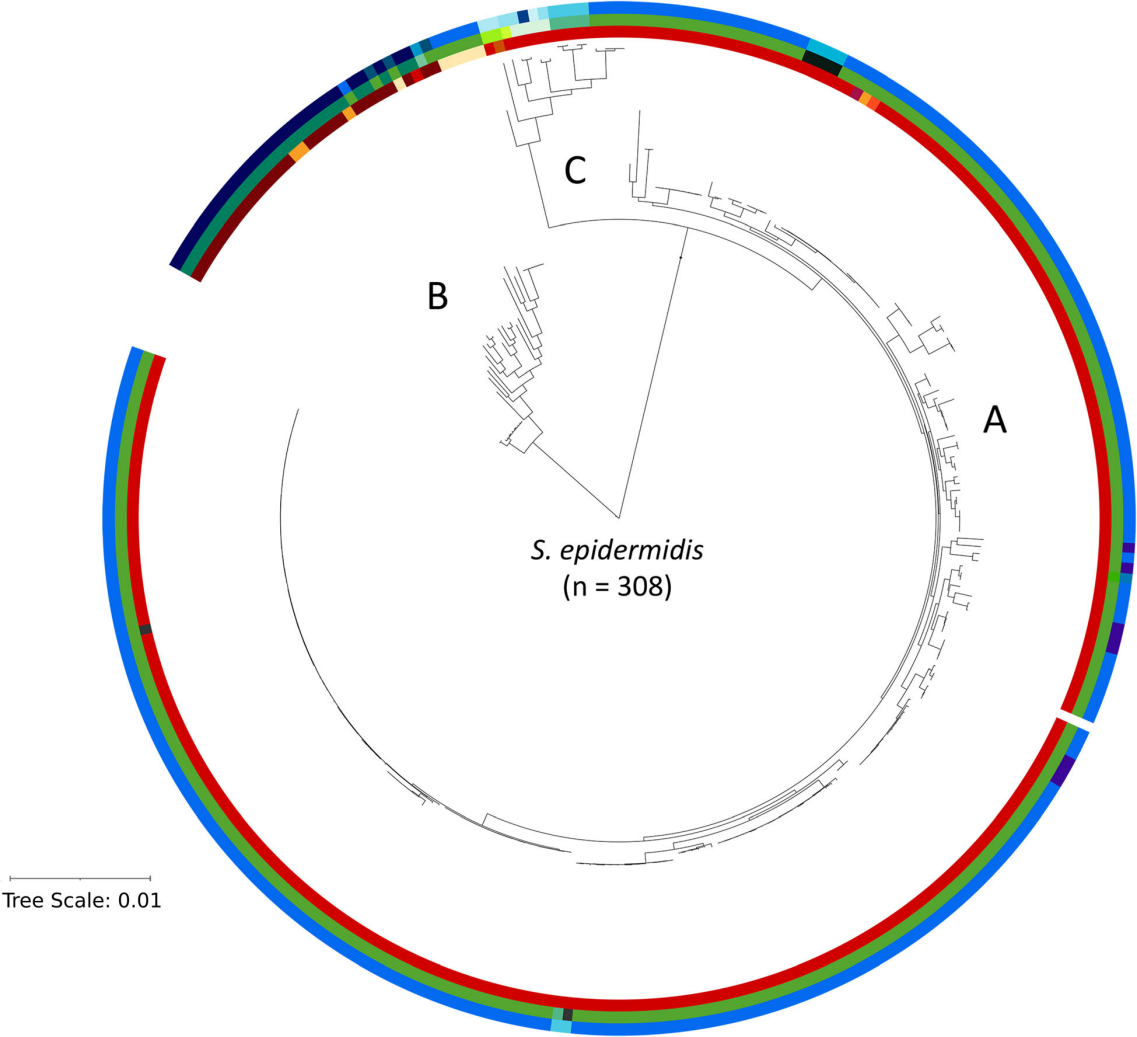
Core genome-based phylogenetic tree of *S. epidermidis* and the resolution power of three amplicon NGS schemes. The phylogenetic tree is based on core genome-SNPs of 308 strains. Different shades of colour represent different alleles of rpsK = red, tuf1 = green and tuf2 = blue, respectively

We observed that three different alleles of *S. epidermidis* were detected with the tuf2 scheme (Fig. 4). Since *S. epidermidis* is the most dominating staphylococcal species on human skin and its phylogenetic diversification into three clades (designated A, B and C) is well reported, we performed a detailed analysis of the resolution power of the three amplicon NGS schemes regarding *S. epidermidis*. A phylogenetic tree based on the core genome of 308 publicly available *S. epidermidis* genomes (Additional file 5) was built and analysed regarding the question whether the amplicon schemes can mirror the population structure. The data showed that the rpsK scheme could unambiguously identify B clade strains of *S. epidermidis*, but it could not distinguish strains of the A and C clades (Fig. 5). The tuf1 and tuf2 schemes could both unambiguously identify most strains regarding their assignment to the phylogenetic clades A, B and C. However, the tuf1 scheme could distinguish less A and B clade strains compared to the tuf2 scheme. Overall, the tuf2 scheme was superior in resolution power.

## Discussion

Staphylococcal populations are an important part of the human skin microbiome and play a central role in health and disease, in a species- and often also in a strain-dependent manner [1–14]. There is a need for efficient tools to determine and discriminate staphylococcal populations on human skin, ideally beyond the species level. Here, we compared three culture-independent amplicon NGS schemes to investigate their suitability and accuracy for analysing staphylococcal populations in human skin samples.

The tuf1 gene fragment was first used for the identification of staphylococcal isolates to the species level [41]. Later, this tuf1 target sequence was applied in an amplicon NGS approach for determining staphylococcal populations of pig skin and pig noses [42]. A modified scheme, relying on a different *tuf* gene fragment (tuf2) was recently developed [47]. In the latter study, samples from healthy skin were analysed, and a surprising finding was the identification of the species *S. saccharolyticus* in relatively high quantities, which was not seen before in other amplicon NGS studies or in culture-dependent studies. In the third scheme analysed here, a *rpsK* gene fragment was used, derived from a prediction with a bioinformatics pipeline [46]. The scheme was used for determining staphylococcal populations of atopic dermatitis affected skin versus healthy skin.

Overall, we could show that all three amplicon NGS schemes accurately identified staphylococcal populations of mock communities as well as of in vivo skin swab samples. All three methods performed comparably well, regardless of DNA input amounts. However, a few differences were detected regarding the detection of *S. saccharolyticus* and different alleles of *S. epidermidis*. First, on the species level, two schemes, the rpsK and tuf1 scheme, were unable to detect *S. saccharolyticus* in all samples, while the tuf2 scheme was able to detect this species. This could be due to a mismatch in the primers to amplify rpsK and tuf1: both reverse primers have one mismatch with the corresponding regions in the genome sequence of *S. saccharolyticus* DVP4-17-2404 and 13 T0028 (data not shown). Second, the tuf1 scheme had problems to accurately detect *S. epidermidis* in both mock communities. A reason could be a mismatch of the reverse tuf1 primer with the corresponding region in the genome sequences of the *S. epidermidis* strains included in mock community M2. Such primer mismatches can lead to an amplification bias and thus an underestimation of the corresponding target [48, 49]. Another reason for the higher resolution power of the tuf2 scheme can also be the amplicon size: it is longer (467 bp), compared to the other two schemes (rpsK = 381 bp; tuf1 = 366 bp). This leads to a better resolution due to a higher number of SNPs in the amplicon, compared to the tuf1 and rpsK amplicons. This is likely the reason why the tuf2 scheme could distinguish more strains in mock community M2 and was able to differentiate between more *S. epidermidis* alleles in in vivo skin samples.

On the other hand, the longer tuf2 amplicon length can create a potential problem, since it leads to fewer usable paired-end reads after quality processing, due to a low sequence quality of reads at the 3’ends with the applied Illumina MiSeq sequencing approach. The analysis of the sequence data obtained with the rpsK and tuf1 schemes showed that a similar portion of paired-end reads passed the quality processing, in average 21.7 and 21.0% respectively (Additional file 6). The proportion of passed reads for the tuf2 scheme was lower compared to rpsK and tuf1 schemes, with an average of 11.6%. This did not seem to have an impact on the quality of results generated in this study, since the mock community populations were best determined by the tuf2 scheme, but could lead to problems when the input DNA concentration of in vivo samples is extremely low.

We further investigated whether the three amplicon NGS schemes can not only detect overall staphylococcal populations on species level, but also whether they can differentiate subspecies and phylogenetic clades of CoNS. Exemplarily, we focused on *S. epidermidis,* because of its abundance on skin and the extensive knowledge about its population structure [24–26]. Previous studies have shown that the population of *S. epidermidis* can be divided in three main clades (A, B and C) [24–26] and that each individual and each skin site is colonised by multiple founder linages of *S. epidermidis* of different phylogenetic clades [50]. In Espadinha et al. [25] it was shown that *S. epidermidis* strains from the A and C clades were more often associated with hospital-infections, while the B clade was mainly associated with commensal *S. epidermidis* strains. We analysed in silico if the schemes can give an accurate picture of the genetic diversity regarding the three phylogenetic clades of *S. epidermidis.* The tuf2 scheme was superior in differentiating the three main clades of *S. epidermidis*, probably due to a longer amplicon sequence and thus higher resolution power. Thus, the tuf2 scheme is able to analyse the presence of each *S. epidermidis* clade and could show which clade is mainly present e.g. in skin samples. However, all schemes including the tuf2 schemes have only limited powers to resolve the population structure of *S. epidermidis*. A species-specific scheme would need to be employed, such as the duplex-amplicon NGS scheme developed by Rendboe et al. [51] to more accurately resolve the diversity of *S. epidermidis.*

Besides the three amplicon NGS schemes analysed here, other studies have used different *tuf* gene fragments for the analysis of staphylococcal populations [43–45]. For example, the *tuf* gene fragment used by McMurray et al. [43] was predicted to distinguish fourteen out of eighteen strains of mock community M2 (data not shown). Two additional *tuf* schemes were developed in the last year [44, 45]; these schemes were predicted to distinguish both 13 strains of mock community M2 (data not shown). Thus, these additional three schemes based on *tuf* gene fragments were predicted to distinguish fewer strains of the mock community M2 than the tuf2 scheme.

The overall limitation of the amplicon NGS schemes analysed here is that they cannot resolve staphylococcal populations to the strain level. This could be achieved by shotgun metagenomic sequencing; however, it relies on a sufficiently high DNA input amount and high sequencing depth and is thus still expensive.

## Conclusion

All three schemes included in this study performed well when analysing staphylococcal populations in mock communities as well as in skin swab samples. However, the tuf2 amplicon NGS scheme determined the expected sample composition best; it could distinguish between more *S. epidermidis* alleles in in vivo samples and detected *S. saccharolyticus* most reliably.

## Methods

### Participants and skin swabbing

Skin swab samples with moistened cotton tips were taken from 13 volunteers (female, *n* = 5; male, *n* = 8) with an age range of 22–43 years from forehead, cheek, back and forearm, as described previously [47]. None of the volunteers had a history of skin disease; none had undergone treatment with topical medicine or antibiotics during the last 6 months. Written informed consent was obtained from all volunteers and the study was approved by the International Medical & Dental Ethics Commission GmbH (IMDEC), Freiburg (Study no. 67885).

### Cultivation of swab sample and species identification

Skin swab samples obtained were diluted in 0.9% NaCl solution and plated out on Columbia agar with 5% sheep blood and cultivated at 37 °C for 24 h. Up to five colonies that resembled staphylococci based on colony size and colour were randomly picked of each plate and pure cultures were obtained by sub-cultivation on the same agar, cultivated at 37 °C for 24 h. Each isolate (254 in total) was assigned to species level by MALDI-TOF mass spectrometry (Additional file 2).

### DNA extraction from skin swab samples

Eight skin swab samples were randomly selected for DNA extraction. Prior to DNA extraction, skin swab samples were centrifuged, and the supernatant was discarded. The pellets were lysed by using lysostaphin (0.05 mg/mL, Sigma) and lysozyme (9.5 mg/mL, Sigma). DNA was extracted using the DNeasy PowerSoil Kit (QIAGEN), following the manufacturer’s instructions. DNA concentrations were measured with the Qubit dsDNA HS Assay (ThermoFisher Scientific) at a Qubit fluorometer.

### Whole genome sequencing

Bacterial isolates were grown on Columbia agar with 5% sheep blood for 24 h at 37 °C. Bacteria were harvested and lysed with lysostaphin (0.05 mg/mL, Sigma). DNA extraction was performed using DNeasy UltraClean Microbial Kit by following manufacturer’s instructions. DNA concentration and purity were measured by Nanodrop. DNA integrity was examined with Genomic DNA ScreenTape (Agilent) at the 4200 TapeStation System. The extracted bacterial DNA was used to generate Illumina shotgun libraries; they were prepared using the Nextera XT DNA Sample Preparation Kit and subsequently sequenced on a MiSeq system using the v3 reagent kit with 600 cycles (Illumina, San Diego, CA, USA) as recommended by the manufacturer. Quality filtering was done with version 0.36 of Trimmomatic [52]. Assembly was performed with version 3.13.0 of the SPAdes genome assembler software [53]. Version 2.2.1 of Qualimap [54] was used to validate the assembly and determine the sequence coverage. Additional file 3 contains information regarding the sequencing and genome statistics, i.e. coverage, contig number, N50 and GenBank accession numbers.

### Bacterial mock communities

Genomic DNA of 18 strains was used to build two mock communities. The DNA was combined in equimolar ratios, with 0.05 ng or 50 ng DNA of each strain. The following bacterial strains were used for two mock communities M1 and M2 (Additional file 1).

The nine-strain-community (M1) contained: *S. epidermidis* ATCC 12228, *S. hominis* HAA31, *S. capitis* HAF22, *S. aureus* DSM 20231, *S. warneri* HAA271, *S. haemolyticus* HAA11, *S. saprophyticus* HAF121, *S. simulans* HAA294 and *S. saccharolyticus* 13 T0028. The 18-strain mixture (M2) contained the same strains as above plus the additional strains: *S. epidermidis* NCIB 11536, *S. epidermidis* HAF81, *S. epidermidis* HAB176, *S. hominis* DSM 20328, *S. hominis* HAB38, *S. capitis* DSM 20325, *S. warneri* DSM 20316, *S. haemolyticus* DSM 20263 and *S. saccharolyticus* DVP4-17-2404. GenBank accession numbers of all genomes of the listed strains are given in Additional file 1.

### Amplicon polymerase chain reaction (PCR) and sequencing

The target fragments, designated tuf1 [41], tuf2 [47], and rpsK [46], were amplified using specific primer sets (Table 1). PCR reaction mixtures were made in a total volume of 25 μl and comprised 5 μl of DNA sample, 2.5 μl AccuPrime PCR Buffer II (Invitrogen, Waltham, MA, USA), 1.5 μl of each primer (10 μM) (DNA Technology, Risskov, Denmark), 0.15 μl AccuPrime Taq DNA Polymerase High Fidelity (Invitrogen, Waltham, MA, USA), and 14.35 μl of PCR grade water. The PCR reaction was performed using the following cycle conditions: an initial denaturation at 94 °C for 2 min, followed by 35 cycles of denaturation at 94 °C for 20 s, annealing at 55 °C for 30 s, elongation at 68 °C for 1 min, and a final elongation step at 72 °C for 5 min. PCR products were verified on an agarose gel and purified using the Qiagen GenereadTM Size Selection kit (Qiagen, Hilden, Germany). The concentration of the purified PCR products was measured with a NanoDrop 2000 spectrophotometer (ThermoFisher Scientific, Waltham, MA, USA). Amplicon NGS was performed as described previously [47].

**Table 1:** Primer sets used in this study (without adapter sequences)

Primer pair name	Amplicon position ^**a**^	Target gene		5′ -- > 3’	Amplicon length [bp]	reference
**rpsK**	2–382	*rpsK*	fw	TGGCACGTAAACAAGTATC	381	[46]
			rev	GACGACGTTTTGGTGGAC		
**tuf1**	688–1053	*tuf*	fw	GGCCGTGTTGAACGTGGTCAAATC	366	[41, 42]
			rev	TIACCATTTCAGTACCTTCTGGTAA		
**tuf2**	685–1151	*tuf*	fw	ACAGGCCGTGTTGAACGTG	467	[47]
			rev	ACAGTACGTCCACCTTCACG		

^a^ Amplicon position in genes (*tuf/rpsK*) of *S. epidermidis* ATCC 12228 (GCA_000007645.1)

### Amplicon NGS data analysis and visualization

FASTQ sequences were processed using QIIME2 (v. 2019.7) [55] as described previously [47]. A cut-off of 99% identity against *tuf* and *rpsK* gene databases was used. The database was build based on all closed staphylococcal genomes available in GenBank (status 02/01/2021). Mock community samples were analysed with a database which contained the rpsK/tuf1/tuf2 allele for each strain. Data was normalized, low abundant features were filtered with a threshold of 2.5%, and figures were prepared in R (v. 4.0.1) with the packages ggplot2 [56] and gplots [57]. Bray-Curtis dissimilarity of mock community sample data was calculated in the vegan package [58] and ordinated in a principal coordinate analysis (PCoA).

### Phylogenomic amplicon target analysis

For phylogenomic analyses, all closed and scaffold genome sequence data of *S. epidermidis* was obtained from NCBI RefSeq (status 07.01.2021). GenBank accession numbers of all used genomes are given in Additional file 5. Genomes were aligned and clustered based on SNPs in their core genome using Parsnp (v 1.0) [59]. The different amplicon alleles were identified for each strain using Blast+ (v 2.11.0). Visualization of the tree was done with iTOL (v 5.7).

## Supplementary Information

**Additional file 1.** Table S1: Composition of two staphylococcal mock communities.

**Additional file 2.** Table S2: Cultivation results of skin swabs from 13 volunteers.

**Additional file 3.** Table S3: Genome statistics of sequenced staphylococcal genomes.

**Additional file 4.** Fig. S1: Phylogenetic trees of the alleles of three amplicon targets (tuf1, tuf2 and rpsK), extracted from 18 staphylococcal genomes present in the M2 mock community.

**Additional file 5 **Table S4: Accession numbers of *S. epidermidis* genomes included in the phylogenomic analysis.

**Additional file 6.** Table S5: Amplicon sequencing statistics of the mock communities.

## Data Availability

Whole genome sequences generated for this project are available in the NCBI BioProject database under the project ID PRJNA702288, and NCBI GenBank accession numbers are listed in Additional file 3. Whole genome sequences of strains used in the mock communities were obtained from NCBI GenBank, and all NCBI GenBank accession numbers are listed in Additional file 1. Amplicon sequencing data are available in the NCBI Sequence Read Archive (SRA) under the project ID PRJNA702649. Whole genome sequences of *S. epidermidis* used for the phylogenomic analysis were obtained from NCBI RefSeq, and NCBI RefSeq accession numbers are listed in Additional file 5.

## Abbreviations

CoNS: coagulase-negative staphylococci
EF-Tu: elongation factor Tu
MALDI-TOF: Matrix-Assisted Laser Desorption – Ionization - Time of Flight
NGS: Next-generation sequencing
PCoA: principal coordinate analysis
PCR: polymerase chain reaction
SNP: single nucleotide polymorphism
SRA: Sequence Read Archive
