# Supplementary material for: Comparison of three amplicon sequencing approaches to determine staphylococcal populations on human skin
Source: BMC Microbiol. 2021 Jul 28;21:221. doi: 10.1186/s12866-021-02284-1 (PMC8320028; doi:10.1186/s12866-021-02284-1)
Supplement: Supplementary file 4 — Additional file 4. Fig. S1: Phylogenetic trees of the alleles of three amplicon targets (tuf1, tuf2 and rpsK), extracted from 18 staphylococcal genomes present in the M2 mock community. [file 12866_2021_2284_MOESM4_ESM.pdf]

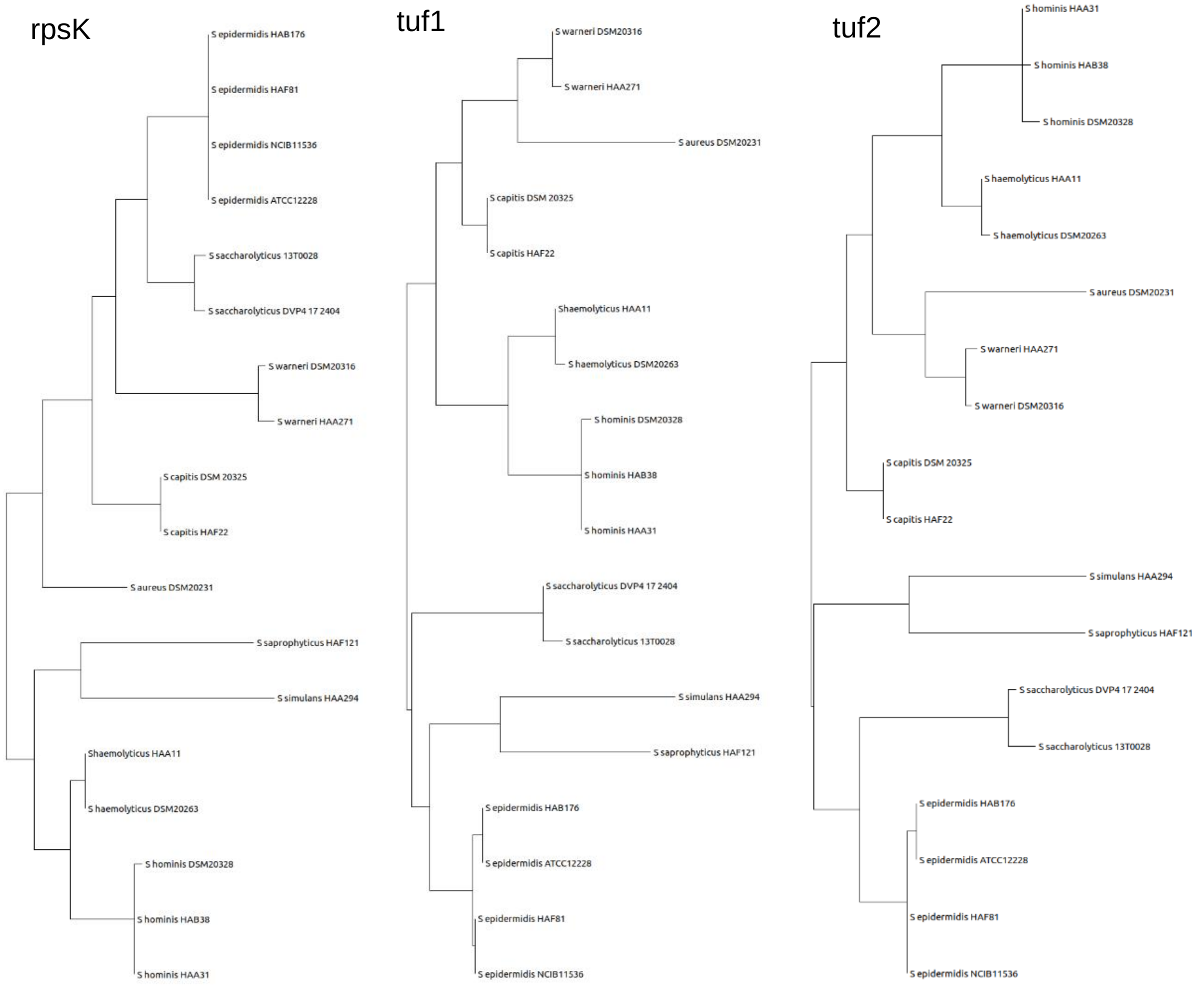

Figure S1. Phylogenetic trees of the alleles of three amplicon targets (tuf1, tuf2 and rpsK), extracted from 18 staphylococcal genomes present in the M2 mock community.
